# The dual role of TRPV1 in peripheral neuropathic pain: pain switches caused by its sensitization or desensitization

**DOI:** 10.3389/fnmol.2024.1400118

**Published:** 2024-09-09

**Authors:** Ning Gao, Meng Li, Weiming Wang, Zhen Liu, Yufeng Guo

**Affiliations:** ^1^Department of Acupuncture and Moxibustion, Guang'anmen Hospital, China Academy of Chinese Medical Sciences, Beijing, China; ^2^Department of Gastroenterology, Guang'anmen Hospital, China Academy of Chinese Medical Sciences, Beijing, China

**Keywords:** TRPV1, peripheral neuropathic pain, molecular mechanisms, sensitization, desensitization

## Abstract

The transient receptor potential vanilloid 1 (TRPV1) channel plays a dual role in peripheral neuropathic pain (NeuP) by acting as a “pain switch” through its sensitization and desensitization. Hyperalgesia, commonly resulting from tissue injury or inflammation, involves the sensitization of TRPV1 channels, which modulates sensory transmission from primary afferent nociceptors to spinal dorsal horn neurons. In chemotherapy-induced peripheral neuropathy (CIPN), TRPV1 is implicated in neuropathic pain mechanisms due to its interaction with ion channels, neurotransmitter signaling, and oxidative stress. Sensitization of TRPV1 in dorsal root ganglion neurons contributes to CIPN development, and inhibition of TRPV1 channels can reduce chemotherapy-induced mechanical hypersensitivity. In diabetic peripheral neuropathy (DPN), TRPV1 is involved in pain modulation through pathways including reactive oxygen species and cytokine production. TRPV1’s interaction with TRPA1 channels further influences chronic pain onset and progression. Therapeutically, capsaicin, a TRPV1 agonist, can induce analgesia through receptor desensitization, while TRPV1 antagonists and siRNA targeting TRPV1 show promise in preclinical studies. Cannabinoid modulation of TRPV1 provides another potential pathway for alleviating neuropathic pain. This review summarizes recent preclinical research on TRPV1 in association with peripheral NeuP.

## 1 Introduction

Neuropathic pain (NeuP) is caused by a lesion or disease affecting the peripheral or central somatosensory nervous system (Baron et al., 2010), as the International Association for the Study of Pain (IASP) defines (IASP, 1979). Depending on the lesion location, NeuP is classified into peripheral and central NeuP according the ICD-11 (Scholz et al., 2019). The prevalence of NeuP is as high as 7–10% of the general population, which is higher in certain specific populations (Torrance et al., 2006; Bouhassira et al., 2008; van Hecke et al., 2014). About 26% of patients with diabetes mellitus and 21% of patients with herpes zoster develop NeuP (van Hecke et al., 2014). The classic symptoms of NeuP involve positive symptoms such as spontaneous pain, hyperalgesia and allodynia, as well as negative symptoms such as decreased or loss of sensation (Scholz et al., 2019; Gilron et al., 2015). Meanwhile, NeuP is often accompanied by different temporal characteristics and pain properties (Gilron et al., 2015). NeuP is typically chronic and severe, impacting patients’ psychosocial and healthcare economic costs as well as their quality of life (Langley et al., 2013; Bates et al., 2019). The management of NeuP is extremely challenging for clinicians due to the refractory treatment (Deng et al., 2016). An epidemiologic survey showed that about 10–20% of patients are not correctly identified (Freynhagen and Bennett, 2009) and about 30–60% are not treated appropriately (Martinez et al., 2014), which may be related to insufficient information about the pathophysiologic mechanisms of the diseases (Moisset et al., 2020). Over the past decades, researchers have begun to investigate the cellular and molecular mechanisms involved in the pathogenesis of NeuP. Studies have revealed that significant mechanisms observed under the NeuP condition, include ectopic activity (Amir et al., 2005), peripheral sensitization (Kiguchi et al., 2014), central sensitization (Koltzenburg et al., 1994), impaired inhibitory regulation (Torsney and MacDermott, 2006), and microglia activation (Thacker et al., 2009).

Transient Receptor Potential (TRP) channels is a non-selective cation channels, consisting of a broad range of channels (Samanta et al., 2018), which could be categorized into TRPC, TRPV, TRPA, TRPM, TRPP, and TRPML (Venkatachalam et al., 2014). The TRP channels are implicated in the transduction of sensory information, including thermosensation (Damann et al., 2008), taste (Dhaka et al., 2006), hearing (Clapham, 2003), pain sensation (Clapham, 2003), etc. The abnormal function of TRP channels may lead to skin (Moran, 2018), airway (Marwaha et al., 2016), endocrine (Brandt et al., 2012) and gut (Cao et al., 2013). Furthermore, TRP channels are essential molecular components in acute inflammation and chronic pain conditions (Liao et al., 2013). Transient receptor potential channel vanilloid subtype 1 (TRPV1) channel is of the most studied targeting mechanisms in NeuP researches due to its widespread expression in neuronal cells and its critical role in pain perception and modulation (Moiseenkova-Bell et al., 2008; Dangi and Sharma, 2024). In this review, we provide a systematic overview of TRPV1, with a particular focus on their role and research progress in NeuP.

## 2 Structure and expression of TRPV1 channel

### 2.1 Molecular structure

TRPV1 was the first mammalian TRP channel whose structure was determined and cloned (Xu et al., 2007). The TRPV1 protein consists of four subunits, each containing six transmembrane structural domains (S1–S6) and two long intracellular N-terminal and C-terminal (Binder et al., 2011). Four independently folded S1–S4 structural domains surround to form the intervening pore loop region, which constitutes an ion-permeable channel with S5 and S6 (Valdes et al., 2011). Single-particle electron cryomicroscopy identified a fourfold symmetric structure of TRPV1, consisting of two regions, a large basket-like domain and a small compact domain, corresponding to the N-terminal, C-terminal region and transmembrane region, respectively (Caterina et al., 1997). Dual gating mechanism regulates the opening of TRPV1, where the upper gate is a selectivity filter formed by a funnel-shaped extracellular pore and the lower gate is located in the middle of the S6 helix and is involved in the dilation of a hydrophobic constriction (Xu et al., 2007). Some studies identified allelic variants of TRPV1 in specific populations (Sondermann, 2019), which may be associated with cold sensitivity (Caterina et al., 2000) and risk of developing knee osteoarthritis (Benítez-Angeles et al., 2020).

### 2.2 Expression patterns in tissues

**Figure 1:**
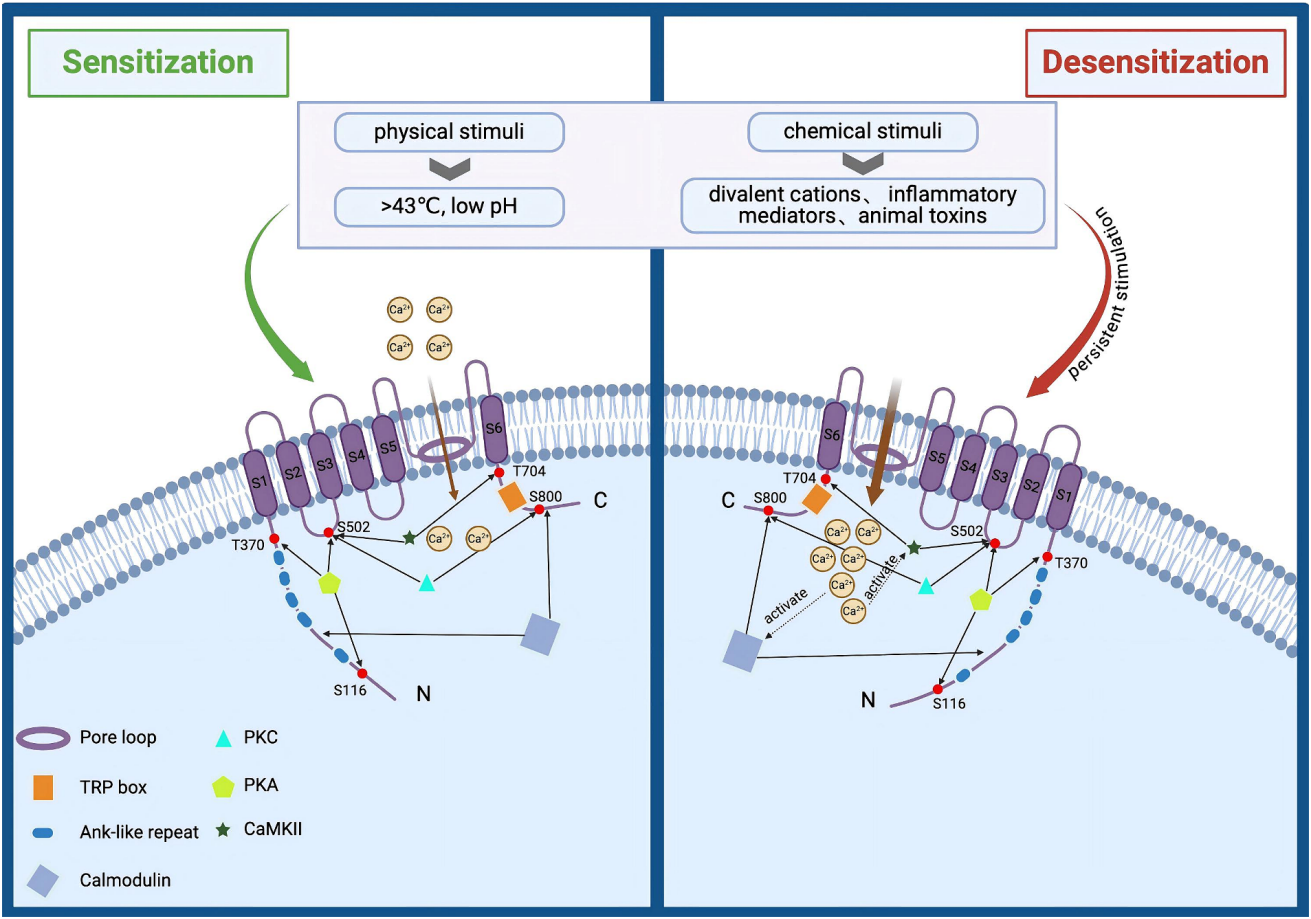
Structural and functional overview of TRPV1 activation and desensitization. TRPV1 is composed of three parts: intracellular N and C termini, six transmembrane domains (S1–S6), and a pore loop region formed between S5 and S6. It can be activated by various physical and chemical stimuli, such as noxious heat (>43°C), divalent cations, low pH, inflammatory mediators, and animal toxins. Activation of this channel leads to significant influxes of Ca^2+^ and Na^+^, causing neuronal depolarization and action potential discharge. Prolonged stimulation enhances TRPV1 activation, reducing neuronal excitability and resulting in near or complete insensitivity to subsequent stimuli, a phenomenon known as specific desensitization. Additionally, elevated intracellular calcium levels can activate the calcium-dependent protease calpain, which degrades cytoskeletal components within axons, leading to axonal structural damage and functional loss.

TRPV1 is abundantly expressed in peripheral sensory neurons of the dorsal root ganglia (DRG), vagus and trigeminal ganglia (Arora et al., 2021). In addition, TRPV1 is also expressed in the intestinal mucosal epithelium, skin epidermis and immune cells, and others (Tominaga et al., 1998). As a pain and heat sensor for humans (Sanz-Salvador et al., 2012), it can be activated by a broad range of physical and chemical stimuli such as toxic heat (>43°C), divalent cations, low pH, inflammatory mediators, and animal toxins (Arora et al., 2021). Activation of the channel leads to a large Ca^2+^ and Na^+^ influx, generating neuronal depolarization and action potential discharges, which may also lead to calcium overload (Sanz-Salvador et al., 2012; Bujak et al., 2019). The activation of TRPV1 is enhanced when multiple stimuli are present simultaneously (Colloca et al., 2017). However, persistent stimulation reduces neuronal excitability, leading to a basic or complete insensitivity to subsequent stimuli, and thus specific desensitization (tachyphylaxis) occurs (Figure 1) (Bujak et al., 2019; Wang et al., 2022). To date, TRPV1 agonists (capsaicin, Resiniferatoxin), as well as antagonists (capsazepine, SB-705498, or NEO6860), have been used for the treatment of migraine, osteoarthritis, atopic dermatitis, and NeuP (Petitjean et al., 2015).

## 3 Mechanisms of NeuP

The pathogenesis of the NeuP is of complexity and has not yet been fully elucidated (Finnerup et al., 2021). Most of the current potential pathogenic mechanisms center on neuronal cells, encompassing the excitability of primary sensory neurons and the imbalance between excitatory and inhibitory synaptic transmission within the central nervous system (CNS) (Nichols et al., 1999). NeuP is typically characterized by ongoing or intermittent spontaneous pain or mechanical allodynia (Willis and Congeshall, 1991). Spontaneous pain may be caused by ectopic activity of damaged nerve fibers, and evoked pain primarily involves peripheral and central sensitization (Inoue and Tsuda, 2018). The reasons for sensitization of nociceptors generally include alteration of ion channels, activation of immune cells, glial-derived mediators, and epigenetic regulation (Inoue and Tsuda, 2018). At the spinal cord level, the underlying synaptic plasticity is not fully clarified. It has been shown that projection neurons in layer I of the spinal dorsal horn form synaptic connections with nociceptor C as well as Aδ fibers. The nociceptive projection neurons in layer I are activated through a complex neural circuit consisting of excitatory and inhibitory interneurons, which then send out projection fibers to carry that stimulus information to the superior centers (Mika et al., 2013; Ji et al., 2019). Peripheral nerve impairment via plastic modification of neuronal synapses and networks leads to changes in the balance between synaptic excitation and inhibition in layer I projection neurons, which may be driven in part by changes in excitatory and inhibitory interneurons in layer II or layer III, that may be related to the development and maintenance of pain hypersensitivity responses (Hains and Waxman, 2006).

The mechanisms of central NeuP involve intricate interactions and maladaptive plasticity within spinal and brain circuits related to nociception and antinociception, along with neuronal hyperexcitability and neuro-immune interactions, contributing to the complexity of this condition (Rosner et al., 2023). Recently, microglia activation was suggested to be involved in central NeuP pathophysiology, leading to the dysregulation of the MED1/BDNF/TrkB signaling pathway within the CNS following thalamic hemorrhage, which in turn induces pain and depression (Infantino et al., 2022). There is also research suggesting that the activation of microglia leads to the reorganization of neural networks within sensory pathways, particularly in the thalamus and primary somatosensory cortex. Microglial depletion can effectively prevent and alleviate mechanical hyperalgesia and abnormal axonal regeneration caused by thalamic hemorrhage (Hiraga et al., 2020).

Glial cells make up about 70% of the total number of cells in the CNS and comprise a variety of cell types including oligodendrocytes, astrocytes, microglia and satellite cells (Wahlman et al., 2018). It has been suggested that microglia and astrocytes are the critical cells that contribute to the development of acute and chronic pain following peripheral and central nerve injury (Old et al., 2015; Ji and Xu, 2021). Microglia and astrocytes respond to peripheral input signals and release proinflammatory mediators (Ji et al., 2018), such as cytokines and chemokines, which can sensitize neurons through activation of their cognate receptors, thereby promoting central sensitization and producing allodynia, hyperalgesia and spontaneous pain (Gim et al., 2011). Microglia are the resident immune cells of CNS and can switch between different activation states in response to various stimuli, primarily classified into M1 (pro-inflammatory) and M2 (anti-inflammatory/repair) (Colton and Wilcock, 2010). Under pathological conditions, microglia often adopt the M1 phenotype, producing pro-inflammatory cytokines such as TNF-α, IL-1β, and IL-6, and releasing reactive oxygen species (ROS) and nitric oxide (NO), thereby exacerbating neuroinflammation and neuronal damage (Correale, 2014). For example, following spinal cord injury, microglia rapidly switch to the M1 state, aggravating tissue damage through the secretion of these inflammatory mediators (Kigerl et al., 2009). In contrast, in the presence of anti-inflammatory signals such as IL-4 and IL-10, microglia can polarize to the M2 phenotype (Tang and Le, 2016). M2 microglia are involved in tissue repair and the resolution of inflammation, producing anti-inflammatory cytokines, promoting the phagocytosis of debris, and supporting neuronal survival and regeneration. The M2 state is crucial during the recovery process following CNS injury (Jin and Yamashita, 2016). Microglia can switch between M1 and M2 states in response to changes in the local microenvironment. Activation of Toll-like receptor 4 (TLR4) drives microglia toward the M1 state, while engagement of anti-inflammatory receptors can induce the M2 phenotype (Zhang et al., 2022). In Neup, the persistent activation of M1 microglia is associated with chronic pain conditions. Pro-inflammatory cytokines released by M1 microglia sensitize pain pathways and maintain the pain state (DeLeo and Yezierski, 2001). Conversely, promoting the conversion to the M2 phenotype has been proposed as a therapeutic strategy to alleviate chronic pain (Song and Suk, 2017; Jin et al., 2020).

In addition, an increasing number of studies have explored the mechanisms of NeuP in terms of altered lipid metabolism of neurolemma, inflammatory cellular glucose metabolism, and glial cellular glucose metabolism in recent years. Studies have shown that nerve injury produces sphingosine-1-phosphate (S1P), and spinal dorsal horn pairs drive NeuP through selective activation of S1P receptor subtype 1 in astrocytes (Hori et al., 2021). Reprogramming of glucose metabolism in microglia promotes the shift of microglia to a pro-inflammatory phenotype as well as increased ROS production. Reprogramming of glucose metabolism in glial cells also contributes to hyperalgesia and allodynia in NeuP (Ramal-Sanchez et al., 2021).

## 4 Functions of TRPV1 in pain regulation

In recent years, TRPV1 ion channels have been increasingly reported to be involved in the regulation of a variety of physiopathological processes in living organisms (Katz et al., 2023; Petroianu et al., 2023; Schumacher, 2010), especially for its role as a crucial mechanism in the development of pain (Ji et al., 2003). TRPV1 receptors are highly expressed mainly on C and some Aδ nociception nerves (nociceptor), is a pivotal molecule in mediating both thermosensory and thermal pain sensitization formation (Arora et al., 2021). Injury leads to activation of TRP nociceptors in the periphery and action potentials are conducted along afferent sensory fibers to dorsal horn synapses. Subsequently, the signal crosses the spinal-thalamic lateral fasciculus, the thalamus, and the sensory cortex of the parietal lobe of the thalamocortex to localize the pain (Wang et al., 2004). Activation of TRPV1 in the periaqueductal gray promotes the release of glutamate, which activates antinociceptive neurons in the rostral ventromedial medulla, thereby modulating pain signal transmission and antinociceptive responses in the CNS (Starowicz et al., 2007).

TRPV1 plays a crucial role in neuroinflammatory responses by sensing stimuli such as high temperatures, acidic environments, and endogenous lipid molecules, leading to calcium influx (Tominaga et al., 1998; Kwon et al., 2021). This activation of sensory neurons results in the release of inflammatory mediators such as calcitonin gene-related peptide (CGRP) and substance P (Herbert and Holzer, 2002). These mediators further activate microglial and astrocytic cells within the CNS, leading to the release of additional pro-inflammatory cytokines such as tumor necrosis factor-alpha (TNF-α), IL-1β, and IL-6, which amplify the inflammatory response and increase pain sensitivity (Vergne-Salle and Bertin, 2021). Additionally, TRPV1 activation exacerbates the inflammatory response through neuro-immune interactions. The inflammatory mediators released by neurons act not only on glial cells but also affect immune cells such as T cells and macrophages (Li and Gupta, 2019). This leads to the aggregation and activation of these cells at the site of inflammation, releasing more inflammatory mediators and further intensifying the inflammation. Persistent TRPV1 activation may also impact the function of the endogenous opioid system, further influencing pain (Zygmunt et al., 1999).

Many endogenous inflammatory mediators (such as prostaglandin E2 and bradykinin, as well as nerve injury factors like nerve growth factor and TNF-α, etc.) have been shown to act directly or sensitize TRPV1 through secondary messengers and/or protein modifications (Premkumar and Ahern, 2000; Ji et al., 2023), leading to allodynia and hyperalgesia (Zhang et al., 2007). TRPV1 sensitization is facilitated by kinases such as protein kinase A (PKA), protein kinase C (PKC), and calcium/calmodulin-dependent protein kinase II (CaMKII) (Wang et al., 2004; Sinharoy et al., 2015). PKC is a prominent participant in pain signaling that can phosphorylate many substrate proteins to regulate the sensitivity of nociceptors (Rathee et al., 2002). PKC regulates the activity of TRPV1 channels mainly through two sites, S502 and S800, and phosphorylation of these two sites sensitizes and facilitates the opening of TRPV1 channels to enable calcium ions to flow into the cell (Numazaki et al., 2002). It was found that PKCε inhibitors completely blocked the enhancement of TRPV1 expression and provided a more significant functional relationship between PKCε and TRPV1 sensitization (Studer and McNaughton, 2010). c-AMP-dependent PKA phosphorylates the n-terminus of TRPV1 (Jung et al., 2004) and regulates channel sensitization directly through the S116, T144, T370, S502, and S800 sites (Sun et al., 2018; Ferreira et al., 2020). Elevated calcium levels in the cell can activate CaMKII, and active CaMKII can directly phosphorylate TRPV1 channels at specific sites Ser 502 and Thr 704 (Anand and Bley, 2011). Dysregulated lipid metabolism may also impact TRPV1 activation or sensitivity, leading to heightened pain signaling and increased pain perception in neuropathic conditions (Szolcsányi, 1993).

It is becoming evident that Botulinum neurotoxins (BoNTs) also regulate the expression and function of TRP channels, which may explain their analgesic effects (Go et al., 2021).

When BoNT-A enters the cell, synaptosomal-associated protein 25 kDa (SNAP25) is cleaved by the protease activity of BoNT-A(1′) (Dong et al., 2006), thereby inhibiting exocytosis. The failure of TRPV1 to translocate to the plasma membrane makes TRPV1 susceptible to ubiquitination and subsequent proteasomal degradation, leading to a decrease in TRPV1 levels, which mediates its antinociceptive effects (Shimizu et al., 2012). Additionally, estrogen and progesterone can influence pain perception by regulating the expression and function of the TRPV1 receptor (Chen et al., 2021; Ortíz-Rentería et al., 2018). Activation of Sig-1R can enhance the sensitization of TRPV1, leading to increased neuronal response to pain stimuli (Zheng and Trudeau, 2023). In females, mechanical pain from paclitaxel-induced CIPN is linked to the IL-23/IL-17A/TRPV1 axis (Luo et al., 2021), while male sensory neurons show greater paclitaxel-induced TRPM8 activity compared to females (Villalba-Riquelme et al., 2022). An increasing number of studies have highlighted the gender dimorphism in chronic pain (Cabañero et al., 2022).

## 5 Role of TRPV1 in mechanisms of NeuP

Hyperalgesia caused by tissue injury or inflammation is typically accompanied with sensitization to TRPV1 channel activity, which is important in the modulation of sensory transmission from primary afferent nociceptors to neurons in the spinal dorsal horn (Xu et al., 2022; Shim et al., 2019). Preclinical models used for peripheral neuropathic pain research commonly include chronic constriction injury (CCI) of the sciatic nerve, diabetic peripheral neuralgia (DPN), chemotherapy-induced neuropathic pain (CIPN), and etc. (Xu and Wang, 2024; Jaggi et al., 2011). The following section primarily focuses on studies based on these various rodent models of peripheral neuropathic pain.

### 5.1 Chemotherapy-induced NeuP

The pathological mechanisms of CIPN may be related to affecting the function of ion channels, signaling by neurotransmitters and neuromodulators, inflammatory mediators, transcription factors (Zajaczkowską et al., 2019), oxidative stress (Zhao et al., 2023), and mitochondrial dysfunction (Chiba et al., 2017). Moreover, structural brain abnormalities, such as axonopathy, small-fiber degeneration, demyelination, and atrophy, are often detected in the peripheral nerves of individuals with CIPN and rodent models of CIPN (Akhilesh et al., 2022). Platinum- and taxane-derived anticancer drugs, induced neurological damage models are widely applied. Spinal cord expression of TRPV1 receptors has been associated with NeuP induced by the aforementioned chemotherapeutic agents (Luo et al., 2018; Son et al., 2021). For instance, Paclitaxel (PTX) induced behavioral hypersensitivity by sensitizing TRPV1 in DRG neurons through TLR4 signaling (Li et al., 2024; Li et al., 2015). TRPV1 has a role in the development of CIPN, and spinal astrocytes and microglia are also engaged in the beginning and maintenance of CIPN (Lee et al., 2021). After the intrathecal injection of the oxaliplatin-treated satellite glial cells-secreted exosomes, mice developed mechanical hypersensitivity, with an increase in the percentage of reactive oxygen species-positive neurons and upregulation of acid-sensing ion channel 3 and TRPV1 expressions in DRG (Luo et al., 2018). TRPV1 is involved in the progression of mechanically allodynia/nociception and thermal hyperalgesia induced by chemotherapeutic agents such as paclitaxel and vincristine (Son et al., 2021). Inhibition of TRPV1 channels suppresses chemotherapeutic agent-induced mechanical hypersensitivity (Li et al., 2015; Chen and Chang, 2019; Oh et al., 2023). Zinc significantly decreased paclitaxel-induced NeuP in mice in a TRPV1-dependent manner (Li et al., 2015), and Decursin promotes the restoration of damaged neuronal networks and inhibits the pain transformation induced by a sudden increase in Ca^2+^ through the inhibition of TRPV1 (Chen and Chang, 2019). The overexpression of TRPV1 in DRG neurons and the pain reaction in paclitaxel-treated rats were significantly reduced by pharmacological blockade of TLR4, which indicates that TRPV1 expression and channel activity in CIPN are regulated by TLR4 (Guo et al., 2019). JI017 alleviates neuralgia by inhibiting TRPV1 expression and the activation of astrocytes in the superficial area of the spinal dorsal horn. However, JI017 only attenuated cold nociception while mechanical nociception remained unchanged, which may be related to its low CNS penetration rate (Oh et al., 2023). Resistance to chemotherapeutic agents and subsequent NeuP are the main factors affecting the course of chemotherapy in patients (Elafros et al., 2022). Chen et al. (2019) discovered that the development of cisplatin resistance is closely linked to the hyperactivation of the epidermal growth factor receptor (EGFR), driven by a transcriptional upregulation of TRPV1 through NANOG. Additionally, TRPV1 facilitates autophagy-mediated EGF secretion via Ca^2+^ influx, which in turn activates the EGFR-AKT signaling pathway, contributing to the acquisition of cisplatin resistance (Yagihashi et al., 2011). In addition, small interfering RNA (siRNA)-based therapeutics targeting TRPV1 has been verified in a number of experiments for the treatment of NeuP, including CIPN (Wang et al., 2012). Experimental studies related to the TRPV1 in the CIPN are presented in Table 1.

**Table 1:** Preclinical evidence relating to TRPV1 and chemotherapy-induced neuropathic pain.

*In vivo* experiment			Cell type	Testing technology	Intervention	References
Animal type	Modeling reagents	Tissue				
C57BL/6 mice	Oxaliplatin (6 mg/kg)	②	–	a, c, d	JI017	Lee et al. (2021)
Male SD rats	Paclictaxel (2 mg/kg)	①, ②	–	a, b, d	Puerarin	Wu et al. (2019)
Male SD rats	Cisplatin (2 mg/kg)	①, ④	–	a, b, c, g	Corydalis saxicola alkaloids	Kuai et al. (2020)
Male SD rats	Paclitaxel (2 mg/kg)	①, ②	–	a, b, c, g	Cinobufacini	Ba et al. (2018)
Male Wistar rats	Paclitaxel (2 or 4 mg/kg)	①	–	a, b, c, d	Ruthenium red+ capsazepine	Hara et al. (2013)
C57BL/6J mice	Paclictaxel (4 mg/kg)	①	HEK293 Cells	a, d, e, i	Zinc acetate	Luo et al. (2018)
C57BL/6J mice	Paclitaxel (2 mg/kg)	–	F11 Cells, HEK293 Cells	b	Decursin	Son et al. (2021)
Male SD rats, ICR mice	Paclictaxel (2 mg/kg)	①, ②, ⑤	RBL-2H3 Cells	a, b, c, g	Quercetin	Gao et al. (2016)
Male Balb/c mice	Vincristine sulfate (75 μg/kg)	–	PC12 Cells	a, c, d, e, g, h	Withametelin	Khan et al. (2021)
Male SD rats	Paclictaxel (2 mg/kg)	①, ②	RBL-2H3 Cells	a, b, f, i	Electroacupuncture	Li et al. (2019)
C57BL/6NJ mice	Oxaliplatin (3 mg/kg)	①, ②	HEK293 Cells	a, c, d, i	–	Rimola et al. (2020)
C57BL/6N mice	Oxaliplatin (3 mg/kg)	①, ②, ③, ④	HEK-293, COS-1 Cells	a, d, e, i	GPR132	Hohmann et al. (2017)
Male BALB/C mice	Oxaliplatin (3.5 mg/kg)	①	HEK293t, K562, LS180, LoVo Cells	a, b, c, d, i	Carbonic anhydrase inhibitors	Potenzieri et al. (2020)
C57BL/6j black mice	Docetaxel (30 mg/kg)	①	SH-SY5Y Cells	a, b, e, g, i	Melatonin, selenium	Ertilav et al. (2021)
NOD-SCID mice	–	–	CaSki, HEK293, Hela, H1299, SiHa, SNU719, AGSGS, SNU668, MKN28, YCC2 Cells	b, c, d, e, f, i	–	Oh et al. (2023)

(1) ①, DRG; ②, spinal cord; ③, sciatic nerve; ④, hind paw skin; ⑤, plasma; ⑥, skin. (2) a, behavioral tests; b, Western blotting; c, immunohistochemistry; d, qRT-PCR; e, electrophysiology; f, immunofluorescence; g, biochemical measurements; h, histopathology; i, calcium imaging. (3) SD, Sprague Dawley; ICR, Institute of Cancer Research; JI017, an herb mixture composed of *Aconitum carmichaelii*, *Angelica gigas*, and *Zingiber officinale*.

### 5.2 Diabetic peripheral neuralgia

Peripheral neuropathy is a common and characteristic complication of diabetes mellitus, causing numbness, tingling, burning pain in the skin, occasionally accompanied by hyperalgesia or allodynia (Mohammadi-Farani et al., 2014). Possible mechanisms of DPN include a vicious cycle involving the production of advanced glycation end products (AGEs), activation of PKC, amplification of the polyol pathway, and excessive release of ROS and cytokines (Carr and Frings, 2019). TRPV1 is linked to diabetes mellitus on multiple fronts, encompassing pancreatic function and insulin secretion, appetite regulation, and energy expenditure or thermogenesis (Zhang et al., 2019). Experimental studies related to the TRPV1 in the DPN are presented in Table 2. Hyperglycemia reduces the expression of cannabinoid receptor-1 (CB1) receptors and increases the expression of TRPV1 receptors in the PC12 cell line, leading to greater toxic effects from TRPV1 activation (Vincent et al., 2007). Enhanced expression of CGRP may promote injured peripheral nerve regeneration, and activated TRPV1 promotes calcium-dependent release of substance P and CGRP in peripheral nerve endings (Chen et al., 2019). Ropivacaine may exacerbate DPN nerve block by inhibiting TRPV1 expression in the dorsal horn, which in turn decreases CGRP release in the spinal cord (Lam et al., 2018). *In vitro*, receptor for advanced glycation end-products (RAGE) expression, signaling, and RAGE-induced ROS production contributed to apoptosis of DRG neurons exposed to high glucose conditions (Roa-Coria et al., 2019). In contrast, RAGE signaling-mediated TRPV1-associated aberrant responses (in terms of cytoplasmic signaling changes including Ca^2+^, PCK, and Src kinases) as well as ROS accumulation directly or indirectly results in TRPV1 function impairment, which are one of the contributing factors to DPN in the diabetic pathologic setting (Abdelkader et al., 2022; Zhang et al., 2020). Sensitization of peripheral TRPV1, TRPA1, and TRPC channels in non-peptidergic fibers by hydrogen sulfide synthesized by the cystathionine β-synthase enzyme, leading to hyperalgesia and loss of peripheral nerve fibers in a rat model of diabetes mellitus, was further validated by local peripheral injections of capsazepine, HC-030031, and SKF-96365 blockers (Agarwal et al., 2020). In addition, using 9-month-old Ins2+/Akita mice, Lam et al. (2018) found that capsaicin activation of TRPV1 in DRG neurons exhibited accelerated current decay, which may provide an explanation for the phenomenon of reduced pain in people with end-stage diabetic peripheral neuropathy in one way. Abdelkader et al. (2022) found that inosine alleviated pain through downregulation of PKC, TRPV1 expression, decreasing Substance P and Transforming growth factor beta in DPN rat model. α-lipoic acid (ALA) may alleviate NeuP in diabetes by regulating TRPV1 expression via affecting NF-κB (Wang Z. et al., 2020). SUMOylation is an important mechanism for protection against endogenous metabolic damage in DPN sensory neurons, and modulation of TRPV1 function through extra-sensory neuronal SUMOylation may yield novel strategies for treating and reversing DPN (Truini et al., 2015).

**Table 2:** Preclinical evidence relating to TRPV1 and diabetic peripheral neuropathy.

*In vivo* experiment			Cell type	Testing technology	Intervention	References
Animal type	Modeling reagents	Tissue				
Male Wistar rats	NA (50 mg/kg), STZ (52.5 mg/kg)	③	–	a, c, d, e, g, h	Inosine	Abdelkader et al. (2022)
Female SD rats	STZ (65 mg/kg)	①	–	a, b, e, f	ALA	Zhang et al. (2020)
Male SD rats, male ICR mice	STZ (55 mg/kg, rats; 150 mg/kg, mice)	①, ②, ⑤	–	a, b, c, g	Berberine	Zan et al. (2017)
Male SD rats	STZ (60 mg/kg)	②, ③	–	a, b, c, e, h	Ropivacaine	Zhang et al. (2019)
Wild type Wistar rats, mini pigs	STZ (55 mg/kg, rats; 150 mg/kg, mini pigs)	⑥	–	a, b, c, g, h	Resiniferatoxin cream	Baskaran et al. (2023)
SNS-Cre mice, Ubc9^fl/fl^ mice	STZ (60 mg/kg)	①, ②, ④	–	a, c, g, i	–	Agarwal et al. (2020)
C57BL/6 mice	25 mM glucose	①	–	b, c, e	Capsaicin	Lam et al. (2018)
Female Wistar rats	STZ (60 mg/kg)	①, ④	–	a, b, c, f	NaHS	Roa-Coria et al. (2019)
C57BL/6J wild-type and Ins2+/Akita mice	5.5 mM glucose	①	–	c, e, i	Capsaicin	Chen et al. (2019)
Male SD rats	High-sugar and high-fat diet, STZ (35 mg/kg)	①	–	a, b, d, f	A438079	Wang et al. (2021)

(1) ①, DRG; ②, spinal cord; ③, sciatic nerve; ④, hind paw skin; ⑤, plasma; ⑥, skin. (2) a, behavioral tests; b, Western blotting; c, immunohistochemistry; d, qRT-PCR; e, electrophysiology; f, immunofluorescence; g, biochemical measurements; h, histopathology; i, calcium imaging. (3) ALA, α-lipoic acid; SD, Sprague Dawley; ICR, Institute of Cancer Research; NA, nicotinamide; STZ, streptozotocin.

### 5.3 Other NeuP

The most common way for creating neuropathy in animals is to cause entire or partial traumatic nerve injury via ligation, transection, or compression (Chen and Pan, 2005). The key protein phospho-regulating effectors that promote nociceptive sensitization are mitogen-activated protein kinases (MAPK), and additional findings showed that baicalin inhibits TRPV1 up-regulation and extracellular signal-regulated kinase phosphorylation in CCI of the sciatic nerve rats’ DRG (Pan et al., 2003). PHN is common in the elderly and immunocompromised patients (Wu et al., 2016). The resiniferatoxin (RTX)-induced PHN model is a commonly used method of PHN modeling, which depletes TRPV1-expressing primary sensory neurons, causing severe degeneration of C-fiber afferent terminals as well as aberrant sprouting of myelinated afferent fibers in the II layer of the spinal dorsal horn (Zhang et al., 2021), which in turn exhibits the distinctive clinical features of PHN, i.e., thermosensory impairments and mechanical allodynia (Story et al., 2003). Wu et al. (2016) proposed that RTX may stimulate the TRPV1 receptor and its downstream signaling molecules to enhance the expression of netrin-1, and the increased expression of netrin-1 further activates repulsive receptor of netrin-1 (UNC5H2) and deleted in colorectal (DCC) at the central terminus of the remaining myelinated neurons in the DRG to promote myelinated fibers to sprout to the noxious neurons located in the superficial dorsal horn. Zhang et al. (2021) found that RTX treatment increased excitatory glutamatergic input from myelinated afferent nerves to the spinal dorsal horn through α2δ-1-dependent enhancement of *N*-methyl-d-aspartate receptor (NMDAR) activity, thereby causing mechanical allodynia, which further enriched the study of synaptic plasticity in PHN. Experimental studies related to the TRPV1 in the other NeuP are presented in Table 3.

**Table 3:** Preclinical evidence relating to TRPV1 and other neuropathic pain.

*In vivo* experiment			Cell type	Testing technology	Intervention	References
Animal type	Modeling reagents	Tissue				
Male SD rats	CCI of the sciatic nerve	①	–	a, b, d	Baicalin	Wang Z. et al. (2020)
Male SD rats	Laminectomy at T10 + Infinite Horizon Impactor	①	–	a, b, e, i	Capsaicin, AMG9810	Wu et al. (2013)
Male Wistar rats	L5 spinal nerve ligation	①, ②, ④	–	a, b, c, d, h	RTX	Javed et al. (2022)
C57bl/6J, TRPV1^Cre^, R26^LSL-tdTomato^, R26^mT/mG^, R26^LSL-hM4Di^ mice	CCI of the infraorbital nerve	–	–	a	Capsaicin, MDL28170	Wang S. et al. (2020)
Male SD rats	Tibial and common peroneal nerves ligation+2–3 mm of the nerve were cut distal to the ligation	①	–	a, b, f, g	CRAP	Li et al. (2017)
Male SD rats	Sciatic nerve ligation	①	–	a, b, c, d	MZF1	Xing et al. (2019)
Male SD rats	RTX (250 μg/kg)	①, ②	SH-SY5Y Cells	a, b, d, f	Capsazepine	Wu et al. (2016)
Mice	RTX (50 μg/kg)	①	–	a, f, h	Adenosine	Kan et al. (2018)

(1) ①, DRG; ②, spinal cord; ③, sciatic nerve; ④, hind paw skin; ⑤, plasma; ⑥, skin. (2) a, behavioral tests; b, Western blotting; c, immunohistochemistry; d, qRT-PCR; e, electrophysiology; f, immunofluorescence; g, biochemical measurements; h, histopathology; i, calcium imaging. (3) SD, Sprague Dawley; CCI, chronic constriction injury; MZF1, myeloid zinc finger 1; CRAP, Coumarins from Radix angelicae pubescentis; RTX, resiniferatoxin.

## 6 Association between TRPA1 and TRPV1

There are evidences that TRPA1 and TRPV1 mutually regulate pain signal transduction (Weng et al., 2015; Spahn et al., 2014). TRPA1 is localized to a subset of TRPV1-positive sensory neurons, being present in 30–50% of these neurons. It is rarely detected in neurons that lack TRPV1 expression (Fischer et al., 2014; Shields et al., 2010). In cells co-expressing TRPA1 and TRPV1, these two TRP channels appear to form a complex or a heterogeneous channel at the cell membrane, thereby influencing the function of each other (Marwaha et al., 2016; Billeter et al., 2014). Shields et al. (2010) utilized selective elimination of the central terminus of TRPV1-expressing nociceptor in wild-type C57Bl/6 mice by intrathecal injection of capsaicin and found that the nociceptive reaction induced by the TRPA1-selective agonist mustard oil was also eliminated. The co-expression of TRPA1 and TRPV1 in nociceptive fibers is crucial for the initiation and progression of chronic pain (Akopian et al., 2007). Structurally, TRPA1 and TRPV1 share similar transmembrane domains. However, TRPA1 differs by having an additional pore helix lining the extracellular side of the ion permeation pathway, resulting in two pore helices per subunit (Mihara and Shibamoto, 2015). Studies of I_Mustard Oil (MO)_ rapid sensitization in Chinese hamster ovary cells expressing TRPA1 or TRPA1/TRPV1 showed that I_MO_ experienced greater rapid sensitization in the absence of TRPV1. One possible explanation is that TRPV1 stabilizes the membrane surface expression of TRPA1 (Fernandes et al., 2012). Activation of TRPA1 did not sensitize TRPV1 without the involvement of calcium ions, suggesting that co-expression occurs in a calcium-dependent way. TRPA1 activation leads to enhanced accumulation of cAMP and subsequent stimulation of PKA subunit release, which in turn leads to phosphorylation and sensitization of TRPV1 (Wu et al., 2019). Functional crossover desensitization has also been reported between typical agonists of TRPA1 (allyl isothiocyanate, mustard) and TRPV1 (capsaicin) (Kuai et al., 2020). In addition, it was shown that TRPA1 and TRPV1 can form complexes in cell membranes that affect the properties of each other (Marwaha et al., 2016). The TRPA1 and TRPV1 channels are therefore described as “partners in crime” (Ba et al., 2018).

## 7 Basic drug targets

### 7.1 TRPV1 agonists

Capsaicin, a potent agonist of the TRPV1 channel, was extracted from the capsicum genus of spices (Abrams et al., 2021). Capsaicin has emerged as a useful tool in the research on pain pathways (Turnbull, 1850) and is currently approved for the treatment of PHN, HIV-associated neuropathy and DPN (Jancso and Jancso, 1949; Szolcsányi, 2005). High concentrations of capsaicin reversibly deactivate TRPV1 receptors, which leads to an analgesic effect (Blair, 2018). It has long been recognized that the initial application of capsaicin is painful and, paradoxically, repeated applications produce local analgesic effects (Touska et al., 2011; Tian et al., 2019). This is a desensitization response induced by prolonged gating of TRPV1 cation channels (Arora et al., 2021) that is closely associated with the duration of capsaicin exposure and the external calcium concentration, and which can be considered as a protective mechanism for neurons against calcium overload during repeated TRPV1 stimulation (Iftinca et al., 2021). Calcium influx following TRPV1 activation leads to channel desensitization. Acute desensitization refers to a rapid decline in the evoked inward current, while tachyphylaxis describes the reduction in current during repeated stimulation (Koplas et al., 1997). Compared to the short-term dysfunction induced by low doses of capsaicin, high doses of capsaicin often elicit dysfunction that lasts for months, which may be related to the structural ablation of TRPV1^+^ nerve endings (Campbell et al., 2021). Capsaicin induces calcium influx through TRPV1 channels, leading to the activation of the calcium-dependent protease calpain. Calpain then begins to degrade cytoskeletal components within the axon, resulting in structural damage and loss of function in the axon. Studies have shown that capsaicin-induced TRPV1^+^ sensory axon ablation is also associated with mitochondrial dysfunction. Inhibiting calcium influx or calpain activity can significantly reduce capsaicin-induced TRPV1^+^ axon ablation (Wang et al., 2017). Calcineurin, also known as protein phosphatase 2B, is a Ca^2+^-Calmodulin (CaM) phosphatase that has been shown to dephosphorylate the channel, thereby promoting its desensitization (Mohapatra and Nau, 2005). Ca^2+^ influx activates phospholipase C (PLC), leading to the depletion of the agonists Phosphatidylinositol 4,5-bisphosphate (PIP2) and Phosphatidylinositol 4-phosphate (PIP). This reduction in PIP2 and PIP levels limits the channel’ s activity, resulting in its desensitization (Lukacs et al., 2007; Lukacs et al., 2013). These findings not only enhance our understanding of the mechanisms behind capsaicin-induced analgesia but also provide a theoretical foundation for improving the use of capsaicin in pain treatment.

### 7.2 TRPV1 antagonists and TRPV1-targeted siRNA

TRPV1 antagonists work by blocking the TRPV1 receptor, preventing calcium influx, and thereby inhibiting the transmission of pain signals. However, the preclinical development of TRPV1 antagonists faces challenges, including potential side effects such as thermoregulation abnormalities (Szelenyi et al., 2004). Thereby, the aim of developing TRPV1 antagonists for pain treatment is to create medications that specifically inhibit the activation of TRPV1 channels by pain-inducing agents, without affecting their activation by thermal stimuli (Chahl, 2024). Subsequently, alternative strategies emerged to target the expression of the TRPV1 channel using genome-editing tools. In a preclinical study, mice treated with TRPV1-targeted siRNA showed a phenotype similar to that of TRPV1 knockout mice (Christoph et al., 2008). Research has shown that paratracheal delivery of TRPV1 siRNA suppresses TRPV1 upregulation in the DRG and spinal cord, effectively eliminating CFA-induced inflammation and chemotherapy-induced thermal hyperalgesia and mechanical allodynia (Kasama et al., 2007). TRPV1 antagonists, including TRPV1 siRNA, have potential roles in the treatment of neuropathic pain (Akhilesh et al., 2022).

### 7.3 Cannabinoid modulation

As an integral part of the extended endocannabinoid system (De Petrocellis et al., 2017), TRPV1 interacts with endocannabinoids through complex molecular mechanisms, thereby regulating the pathophysiological processes of neuropathic pain (Starowicz and Przewlocka, 2012). Firstly, TRPV1 can directly interact with endocannabinoids. For example, Anandamide (AEA) is not only a partial agonist of CB1 receptors but also an agonist of TRPV1. When AEA binds to TRPV1, it leads to the opening of TRPV1 channel, causing Ca^2+^ influx, which subsequently induces depolarization and the generation of action potentials in sensory neurons (Fenwick et al., 2017). Secondly, the endocannabinoid system can influence the occurrence and development of neuropathic pain by regulating TRPV1 expression and function. Studies have found that activation of CB1 receptors can inhibit TRPV1 expression and function. For example, treatment with CB1 receptor agonists can reduce TRPV1 expression in sensory neurons, thereby alleviating pain (McDowell et al., 2013). This mechanism may be achieved by lowering intracellular cAMP levels and inhibiting PKA activity, which in turn reduces the transcription and translation of the TRPV1 gene (Vetter et al., 2006). Additionally, TRPV1 may be involved in the degradation process of endocannabinoids. The degradation of endocannabinoids primarily relies on the enzyme fatty acid amide hydrolase (FAAH) (Vandevoorde and Lambert, 2005). For instance, research indicates that increasing doses of a locally injected FAAH inhibitor elevate spinal AEA levels, which in turn produce anti-hyperalgesic and anti-allodynic effects. These effects are achieved through mechanisms that progressively involve the desensitization of TRPV1 channels (Starowicz et al., 2013).

## 8 Conclusion and perspectives

TRPV1 plays a dual role in peripheral NeuP, acting as a “switch” for pain through its sensitization and desensitization processes. In CIPN and DPN, the sensitization of TRPV1 channels is a key mechanism. Inhibiting TRPV1 channels can significantly reduce mechanical hypersensitivity and pain. Clinically, capsaicin, a TRPV1 agonist, alleviates pain by inducing receptor desensitization, while TRPV1 antagonists and siRNA targeting TRPV1 show promise in preclinical studies. Cannabinoid modulation of TRPV1 offers another potential pathway for alleviating neuropathic pain. Future research should focus on the immunomodulation and metabolic functions of the TRPV1 receptor, as well as the application of novel gene editing and RNA interference technologies, with the aim of developing more effective pain treatment strategies.

## Glossary

**Table:**

AGEs	advanced glycation end products
AEA	anandamide
ALA	α-lipoic acid
BoNTs	Botulinum neurotoxins
CaMKII	calcium/calmodulin-dependent protein kinase II
CB1	cannabinoid receptor-1
CCI	chronic constriction injury
CGRP	calcitonin gene-related peptide
CHO	Chinese hamster ovary
CIPN	chemotherapy-induced neuropathic pain
CRAP	Coumarins from Radix angelicae pubescentis
CNS	central nervous system
CaM	*N*-methyl-d-aspartate receptor
DCC	deleted in colorectal
DPN	diabetic peripheral neuralgia
DRG	dorsal root ganglion
DCC	deleted in colorectal
EGFR	epidermal growth factor receptor
FAAH	fatty acid amide hydrolase
IASP	International Association for the Study of Pain
ICR	Institute of Cancer Research
MAPK	mitogen-activated protein kinases
MZF1	Myeloid zinc finger 1
NA	nicotinamide
NO	nitric oxide
NMDAR	*N*-methyl-d-aspartate receptor
NeuP	Neuropathic pain
PHN	postherpetic neuralgia
PTX	Paclitaxel
PLC	phospholipase C
PIP2	Phosphatidylinositol 4,5-bisphosphate
PIP	Phosphatidylinositol 4-phosphate
PKA	protein kinase A
PKC	protein kinase C
RAGE	receptor for advanced glycation end-products
ROS	reactive oxygen species
RTX	Resiniferatoxin
S1P	sphingosine-1-phosphate
SD	Sprague Dawley
siRNA	small interfering RNA
STZ	streptozotocin
TRP	transient receptor potential
TRPV1	transient receptor potential vanilloid 1
TLR4	Toll-like receptor 4
TNF-α	tumor necrosis factor-alpha
